# Investigating patients’ preferences for new anti-diabetic drugs to inform public health insurance coverage decisions: a discrete choice experiment in China

**DOI:** 10.1186/s12889-022-14244-z

**Published:** 2022-10-05

**Authors:** Jinsong Geng, Haini Bao, Zhe Feng, Jingyi Meng, Xiaolan Yu, Hao Yu

**Affiliations:** 1 Medical School of Nantong University, 226001 Nantong, Jiangsu China; 2 The First People’s Hospital of Lianyungang, 222061 Lianyungang, Jiangsu China; 3 Department of Population Medicine, Harvard Medical School and Harvard Pilgrim Health Care Institute, 02215 Boston, MA USA

**Keywords:** Patients’ preferences, Health insurance, Anti-diabetic drug, Discrete choice experiment

## Abstract

**Background:**

Diabetes is a major public health concern with a considerable impact on healthcare expenditures. Deciding on health insurance coverage for new drugs that meet patient needs is a challenge facing policymakers. Our study aimed to assess patients’ preferences for public health insurance coverage of new anti-diabetic drugs in China.

**Methods:**

We identified six attributes of new anti-diabetic drugs and used the Bayesian-efficient design to generate choice sets for a discrete choice experiment (DCE). The DCE was conducted in consecutive samples of type 2 diabetes patients in Jiangsu Province. The mixed logit regression model was applied to estimate patient-reported preferences for each attribute. The interaction model was used to investigate preference heterogeneity.

**Results:**

Data from 639 patients were available for analysis. On average, the most valued attribute was the improvement in health-related quality of life (HRQoL) (β = 1.383, p < 0.001), followed by positive effects on extending life years (β = 0.787, p < 0.001), and well-controlled glycated haemoglobin (β = 0.724, p < 0.001). The out-of-pocket cost was a negative predictor of their preferences (β = -0.138, p < 0.001). Elderly patients showed stronger preferences for drugs with a lower incidence of serious side effects (p < 0.01) and less out-of-pocket costs (p < 0.01). Patients with diabetes complications favored more in the length of extended life (p < 0.01), improvement in HRQoL (p < 0.05), and less out-of-pocket costs (p < 0.001).

**Conclusion:**

The new anti-diabetic drugs with significant clinical effectiveness and long-term health benefits should become the priority for public health insurance. The findings also highlight the value of accounting for preference heterogeneity in insurance policy-making.

**Supplementary Information:**

The online version contains supplementary material available at 10.1186/s12889-022-14244-z.

## Background

Diabetes is a group of metabolic disorders characterized by hyperglycemia resulting from defects in insulin secretion, insulin action, or both [1]. Diabetes imposes a heavy burden on public health. Global diabetes prevalence in 2019 was estimated to be 9.3% (463 million people), rising to 10.2% (578 million) by 2030 and 10.9% (700 million) by 2045 [2]. Over time, diabetes can lead to multiple serious long-term complications such as kidney failure, blindness, heart attacks, stroke, and lower limb amputation [3]. Furthermore, diabetes and its complications impose significant economic impacts on individuals and their families, health systems, and national economies [4]. Most patients need to take anti-diabetic drugs for their whole lives to stabilize and control their blood glucose levels. In recent years, newer anti-diabetes drugs have been developed, not only helping reduce blood glucose levels but also helping slow or prevent the progression of the disease [5].

In China, there are currently over 114 million people with diabetes [6]. The estimated diabetes-related health expenditures in China reached USD 109.0 billion in 2019, posing a massive challenge to the country’s health insurance system [4]. To alleviate the financial burden of patients and boost the utilization of necessary healthcare services, China achieved universal health insurance coverage in 2011 with 95% of its population covered by public health insurance programs [7]. However, gaps remain in the quality of care, control of chronic diseases, control of health expenditures, and public satisfaction [8]. To enhance equity and improve efficiency, patient values need to be considered in the determination of health insurance coverage scope [9].

Nowadays, one of the guiding principles of China’s public health insurance programs is to make Chinese have a greater sense of fulfillment, happiness, and security [10]. Public health insurance programs in China adhere to the people-centered approach and try to meet the reasonable medication needs of the insured [11]. Patient preference is also an essential element in the benefit-risk assessment of new drugs in China [12]. Taking into account the patient voice in health insurance decision-making can result in reimbursement of health technologies that are accepted by patients [13, 14]. Patients would be satisfied with health insurance schemes if the reimbursed health technologies meet their preferences [15, 16]. Nevertheless, direct patient involvement was thought to be subjective, potentially biased, and lacking representativeness [17, 18]. To explore the patient voice in a robust manner, it is necessary to quantify patients’ preferences before they can be adequately considered [18]. As shown in a systematic review, patients’ preferences could be quantitatively generated to inform health insurance decisions [19].

A key component of the evidence base that can guide decision-making on public health insurance coverage is patient values. Recognition of patient values has led to a shift in health technology assessment (HTA) from only looking at clinical outcomes to taking into account the patients’ perceptions of how these outcomes are related to their lives [20, 21]. Incorporating patients’ views into healthcare decisions improves patient satisfaction while fostering healthcare services more strongly aligned with their preferences and expectations [22, 23]. Previous studies in foreign countries showed successful experiences of integrating patient and public preferences into health insurance coverage decision-making [24–27]. However, the assumption of homogeneity in preferences across individuals can lead to misleading policy conclusions [28]. Accounting for preference heterogeneity is important in order to obtain unbiased estimates.

One well-established quantitative technique to elicit stated preferences is the discrete choice experiment (DCE), which allows participants to choose the alternative that maximizes their utility. By observing trade-offs as the participants accomplish a series of choice tasks, DCEs were able to predict choices-mimicking real-world decisions [29, 30]. Previous DCEs on diabetes patients’ preferences mainly focused on therapeutic interventions in clinical settings and several attributes of anti-diabetic drugs were identified, such as the chance of reaching the target glycated haemoglobin (HbA_1c_) level [31, 32], risk of hypoglycemia [31], risk of gastrointestinal problems [32], mode of drug administration [33], out-of-pocket costs, and life expectancy [34]. Health-related quality of life (HRQoL) is an important health outcome, representing one of the major goals of health interventions. To the best of our knowledge, HRQoL was seldom used as an attribute to investigate patients’ preferences for anti-diabetic drugs.

Health insurance decision-making is a complicated process. Few studies to date have examined whether diabetic patients’ preferences for insurance coverage varied according to their demographic features. Diabetes can lead to the development of chronic complications, which increase the disease severity [35, 36]. Despite having the highest prevalence of diabetes of any age group, older patients and/or those with complications have often been excluded from clinical trials [37]. Therefore, decision-makers did not have sufficient evidence regarding patient values of new treatment options for the population. In addition, diabetes poses economic burdens on patients and their families. Previous studies in China showed that low-income patients were more likely to experience catastrophic health expenditure as a result of diabetes care [38]. However, the impact of income on patients’ preferences for insurance coverage of new anti-diabetic drugs was rarely validated.

The assessment of patients’ preferences is necessary to understand the demand for insurance reimbursement. To expand the existing knowledge in this area, our DCE aimed to determine the relative importance of attributes of new anti-diabetic drugs for health insurance coverage from patients’ perspectives. We hypothesized that attributes relevant to health benefits might have the highest ranking, and patients’ preferences differed by age, level of income, and whether they had diabetes complications.

## Methods

### Identification of technology attributes and levels

We took a three-step approach in the preliminary stage of DCE, which aimed to define the attributes and levels of new anti-diabetic drugs. First, a literature review was conducted to identify attributes that were used in previous DCEs on diabetes therapy, management, and policy-making. Data extraction form of attributes and levels was developed according to the EVIDEM framework for integration of evidence and value in decision-making [39, 40]. A flow diagram of the identification of included studies is shown in Appendix 1. Our review found that the attributes mainly involved effectiveness, safety/tolerability, convenience, economic consequence, and patient-reported outcomes (Appendix 2).

Second, since the universe of attributes was vast, focus group discussions with physicians, health insurance decision-makers, and healthcare experts were carried out to determine the attributes. To better define the levels of attributes, we also searched the widely used HTA database established by the National Institute for Health and Care Excellence and the Canadian Agency for Drugs and Technologies in Health, then identified HTA reports for the new drugs treating diabetes. We found 47 reports which had been published before 19th August 2020 and analyzed the attributes and levels of new anti-diabetic drugs.

Finally, a pilot survey with 116 type 2 diabetes patients was conducted to provide feedback on the intelligibility and acceptability of the questionnaire. Responses from the patients led to a more explicit and apprehensible statement of the survey questions. Attributes and levels of new drugs used in our research are listed in Table 1. In this study, we defined new anti-diabetic drugs as the drugs which had been marketed in China for the treatment of diabetes but were not covered by the public health insurance programs. The explanations of attributes and levels are shown in Appendix 3.

**Table 1:** Attributes and levels of new anti-diabetic drugs in the DCE

Domains	Attributes	Levels	Coding
Effectiveness	HbA_1c_ control	Not as expected; As expected	Binary
	Length of extended life	0.5 year to 3.5 years	Continuous
Safety/tolerability	Serious side effects	Sometimes; Occasionally; Never or rarely	Categorical
Patient-reported outcomes	Change in HRQoL	Worse; No improvement; Improvement	Categorical
Convenience	Dosing frequency	Twice a day; Once every other day; Once a week	Categorical
Economic consequences	Out-of-pocket costs per month (if the drug is covered by the insurance program)	CNY 100 to 500^#^	Continuous

HbA_1c_: glycated haemoglobin; ^#^The average exchange rate between US Dollars and the Chinese Yuan (CNY) in 2020 was 1: 6.90. CNY 100 was approximately US$14.49 and CNY 500 was about US$72.49.

### Experimental design and development of the questionnaire

We used Ngene1.2 software (Choice-Metrics, Sydney, Australia) to implement the Bayesian D-efficiency experimental design. The blocking technique was conducted to promote response efficiency by reducing the potential cognitive burden on respondents [41]. Our experimental design comprised 48 pairs of scenarios split into six blocks. The final scenarios with orthogonality, attribute level balance, partial attributes or levels overlap and utility balance was drawn from a series of candidate scenarios [42], and each respondent was required to complete eight pairs of choice tasks.

Given the fact that there was no general standard on the ideal sample size required for a DCE [43], we followed a rule-of-thumb [44] for determining the sample size:

$$\frac{nta}{c}\ge 500$$

where *n* was the number of respondents, *t* was the choice pairs for each block, *a* was the number of alternatives, and *c* equaled the largest number of levels for any attributes. Therefore, the minimum sample size for each block was 94, which was equivalent to a total sample size of 564.

We adopted unlabeled DCE, which had been widely used to explore patients’ preferences for health technologies [34, 45–47]. Respondents of unlabeled DCEs were not subject to the psychological cues of the technology labels, thus reflecting the real decisions [48]. In addition, we applied the forced-choice sets since when no option had a definitive advantage, forced-choice under preference uncertainty led to the selection of options that were relatively easy to justify and associated with a lower chance of error and regret [49]. Examples of choice scenarios are shown in Appendix 4.

Our questionnaire consisted of three parts. The first part included patients’ socioeconomic characteristics, medical history, and complications. The second part contained the DCE tasks. The final part presented patients’ comprehension and confidence when making DCE choices. The patients were asked to rate their own comprehension and confidence on a scale ranging from zero (worst case) to 10 (best case) (Appendix 5).

### DCE implementation and data collection

The formal DCE was conducted from 9th November 2020 to 6th January 2021. Inclusion criteria were patients aged 18 years or older, participating in a health insurance program, diagnosed with type 2 diabetes for at least one year, and taking medications regularly. Patients were excluded if they had been diagnosed with gestational diabetes. There were nine sampling hospitals in four cities (i.e., Suzhou, Nantong, Yancheng, and Lianyungang) in Jiangsu Province. To ensure the representativeness of patients, the sampling hospitals consisted of tertiary, secondary, and primary hospitals. Patients were enrolled consecutively within each hospital.

To ensure the reliability and validity of the survey, our DCE questionnaires were administered via one-to-one, face-to-face interviews. Our interviewers comprised 10 medical interns and 27 physicians. For quality assurance, we compiled a survey manual and trained the interviewers before the experiment. Interviewers were trained on a one-to-one basis, either face-to-face or online, to make them fully comprehend the requirements of the survey. To assure completeness of the questionnaire, the interviewers were required to check each questionnaire immediately after the survey was completed. For patients who were illiterate or had blurred vision, the interviewers explained each item of the questionnaire in detail until the patients fully understood. Medical history and clinical information like complications in the questionnaire were checked from electronic medical records.

We supposed that due to the constrained budget of public health insurance, only one drug could be reimbursed. Patients were asked to think carefully and make trade-offs between the new drugs. Each survey took 20 min to one hour. Patients’ participation in the survey was anonymous and voluntary, and their informed and verbal consent was obtained prior to the survey.

### Statistical analysis

Our DCE data analysis was based on the random utility maximization theory [50, 51]. The utility (U) that patient, i, assigned to choice, j, consists of two parts [42]. One is the observable component *V*_*ij*_, which is determined by patients’ preferences for attributes. The other is the random component *ε*_*ij*,_ representing the random error term with standard statistical properties. Therefore, the utility that a patient gets from choices can be expressed in the following equations:

$${U}_{ij}={V}_{ij}+{\epsilon }_{ij}={\beta }_{0}+{\beta }_{1}{X}_{1ij}+{\beta }_{2}{X}_{2ij}+\dots +{\beta }_{m}{X}_{mij}+{\epsilon }_{ij}$$

where *β* quantifies the strength of preference for each attribute level [42]. Each estimated coefficient is a preference weight and represents the relative contribution of the attribute level to the utility that respondents assigned to an alternative.

We implemented the above equation by mixed logit regression using STATA 14.2 SE (STATA Corp LLC, College Station, Texas, USA). Maximum simulated likelihood leads to the reasonable accuracy of estimation results [52, 53]. Therefore, we first specified the mixed logit model with 500, 1000, 1,500, and 2,000 Halton draws respectively. After that, we selected the specification with 1,500 Halton draws due to the maximum simulated likelihood estimation of the model. Based on effects coding, a positive coefficient indicated that patients would prefer this attribute level compared to the mean effect, while a negative coefficient showed that patients would prefer this level less than the mean effect [54]. The relative importance (RI) of each attribute was calculated based on the overall utility value of the attribute (i.e., the differences between the highest and lowest coefficients of each attribute) divided by the sum of overall utility values across all attributes [55, 56].

To go further into the assessment of preference heterogeneity, we established models that included interaction terms between individual-specific characteristics and attributes, as suggested by Umar et al. [57]. The identified interaction terms were drug attributes with age, level of income, and diabetes complications. The mixed logit regression model that involved interaction terms was as follows:

$$\eqalign{{U}_{ij} & = {\beta }_{0}+{\beta }_{1}{X}_{1ij}+{\beta }_{2}{X}_{2ij}+\dots \cr & +{\beta }_{m}{X}_{mij}+{\beta }_{s1}{X}_{1ij}{S}_{interaction\_term}\cr & +{\beta }_{s2}{X}_{2ij}{S}_{interaction\_term}+\dots \cr & +{\beta }_{sm}{X}_{mij}{S}_{interaction\_term}{+ \epsilon }_{ij}}$$

where *β*_*1−*_*β*_*m*_ quantified the strength of preference for each attribute, *β*_*s1−*_*β*_*sm*_ represented the parameter weights for interaction terms, and *X*_*mij*_*S*_*interaction_term*_ was the interaction terms. The Chi-square test for joint significance was performed to evaluate whether preferences varied. The statistically significant interaction effects would indicate that patients’ preferences differed by specific characteristics [58]. The positive coefficients of interaction terms suggest that compared with a subgroup of patients without a certain characteristic, the subgroup of patients with the characteristic attached more importance to an attribute [59]. While the negative coefficients of interaction terms suggest that the subgroup of patients with a certain characteristic attached less importance to an attribute than the comparators [59].

## Results

### Patients’ characteristics

A total of 670 patients consented to participate in our DCE survey. Of them, 31 were excluded from the analysis due to incomplete data, a lack of understanding, or no confidence in making DCE choices. As a result, data from 639 patients were available for analysis. The average age of the patients was 65.59 years old. 344 patients (53.83%) had monthly household income equal to or less than CNY 6000. 306 patients (47.89%) had diabetes complications. On average, the study patients found it easy to understand the scenarios (8.31, 95% CI 8.25–8.36), and confident in their choices (9.17, 95% CI 9.10–9.24). Further demographic characteristics of the patients are presented in Table 2.

**Table 2:** Demographic characteristics of the study patients (N = 639)

Characteristics	N (%)
Gender	
Male	342 (53.52)
Female	297 (46.48)
Age	
< 65	266 (41.63)
65–74	241 (37.71)
≥ 75	132 (20.66)
Education	
Unschooled	121 (18.94)
Elementary school	195 (30.52)
Junior high school/High school	255 (39.90)
Junior college or above	68 (10.64)
Occupation	
Farmer	264 (41.32)
Urban employee	143 (22.38)
Retiree	156 (24.41)
Unemployed and freelancers	76 (11.89)
Monthly household income (CNY)	
≤ 2,000	88 (13.77)
2,001 ~ 4,000	126 (19.72)
4,001 ~ 6,000	130 (20.34)
6,001 ~ 8,000	98 (15.34)
8,001 ~ 10,000	60 (9.39)
10,001 ~ 12,000	43 (6.73)
> 12,000	94 (14.71)
Complications	
With	306 (47.89)
Without	333 (52.11)

### Main model estimation of preferences

As shown in Table 3, HbA_1c_ control, the incidence of serious side effects, length of extended life, change in HRQoL, dosing frequency, and out-of-pocket costs significantly influenced patients’ new drug choices for insurance coverage. The relative importance, based on the ranking of attribute coefficients for highest versus lowest levels, showed that change in HRQoL was the most important consideration, followed by the length of extended life, HbA_1c_ control, out-of-pocket costs, the incidence of serious side effects, and dosing frequency. The most important attribute level was significant improvement in HRQoL (β = 1.383, p < 0.001), followed by longer extended life (β = 0.787, p < 0.001), and well-controlled HbA_1c_ (β = 0.724, p < 0.001). On the other hand, the out-of-pocket cost was a negative predictor of their preferences (β = -0.138, p < 0.001).

**Table 3:** Estimation of preferences from the mixed logit model

Attributes	β coefficients			RI
	Mean (SE)	SD (SE)		
HbA_1c_ control				16.24
Not as expected (ref)	-0.724^***^(0.073)			
As expected	0.724^***^(0.073)	0.825^***^(0.102)		
Serious side effects				7.39
Sometimes (ref)	-0.377^***^(0.072)			
Occasionally	0.096 (0.061)	-0.031 (0.135)		
Never or rarely	0.281^***^(0.061)	-0.235 (0.208)		
Length of extended life				26.48
Extended life (per year)	0.787^***^(0.074)	0.991^***^(0.091)		
Change in HRQoL				34.25
Worse (ref)	-1.670^***^(0.124)			
No improvement	0.287^***^(0.070)	-0.432^**^(0.138)		
Improvement	1.383^***^(0.110)	1.121^***^(0.109)		
Dosing frequency				3.27
Twice a day (ref)	-0.132^*^(0.056)			
Once every other day	-0.028 (0.060)	0.423^***^(0.101)		
Once a week	0.160^**^(0.061)	-0.202 (0.183)		
Out-of-pocket costs per month				12.38
Cost (per CNY50)	-0.138^***^ (0.021)	0.327^***^ (0.030)		
Log likelihood	-2600.171			
Participants	639			
Observations	10,224			

RI, relative importance; Ref, reference; SE, standard error; SD, standard deviation; ^*^p < 0.05; ^**^p < 0.01; ^***^p < 0.001. Each participant completed eight pairs of DCE choice scenarios, and the number of observations per participant was 16. So there were 10,244 observations for 639 participants

### Interaction-effects model estimation of preference heterogeneity

The model estimation of the interaction effects with each attribute is listed in Table 4. Compared with patients who were less than 65 years old, elderly patients showed stronger preferences for drugs with a lower incidence of serious side effects (β = 0.358, p < 0.01) and less out-of-pocket costs (β= -0.107, p < 0.01) (Model 1). While young or mid-aged patients would be more likely to choose drugs with the lowest incidence of serious side effects (β= -0.333, p < 0.01).

**Table 4:** Estimation of preference heterogeneity from interaction-effects models

Interaction terms	Age (Model 1)			Income (Model 2)			Diabetes Complications (Model 3)	
	Mean	SE		Mean	SE		Mean	SE
Improvement in HbA_1c_								
As expected	0.005	0.121		0.542^***^	0.136		0.152	0.141
Serious side effects								
Occasionally	0.358^**^	0.125		-0.001	0.124		0.184	0.129
Never or rarely	-0.333^**^	0.116		0.025	0.117		-0.193	0.124
Extended life								
Extended life (per year)	0.030	0.122		0.122	0.132		0.404^**^	0.141
Change in HRQoL								
No improvement	0.178	0.131		-0.103	0.139		-0.208	0.142
Improvement	0.249	0.184		0.145	0.172		0.402^*^	0.200
Dosing frequency								
Once every other day	0.076	0.121		0.126	0.123		0.106	0.126
Once a week	0.027	0.128		0.160	0.126		-0.195	0.133
Out-of-pocket costs per month								
Cost (per CNY50)	-0.107^**^	0.040		-0.175^***^	0.042		-0.156^***^	0.043
Log likelihood	-2586.191			-2580.122			-2586.387	
Observations	10,224			10,224			10,224	

Age, income, and diabetes complications were treated as categorical variables in interaction-effects models. Age: Young and middle-aged = 0, Elderly = 1; Income: More than CNY 6000 = 0, CNY 6000 and below = 1; Diabetes complications: Without = 0, With = 1; SE, standard error; ^*^p < 0.05; ^**^p < 0.01; ^***^p < 0.001

Similarly, we tested for interactions of income with the attributes. During the decision-making process, low-income patients conferred much more importance on out-of-pocket costs (β= -0.175, p < 0.001) and the well-control of HbA_1c_ (β = 0.542, p < 0.001) than the high-income patients (Model 2).

The interaction terms regarding diabetes complications with three attributes were statistically significant (Model 3). Compared with patients without complications, patients who had complications favored more in the length of extended life (β = 0.404, p < 0.01), improvement in HRQoL (β = 0.402, p < 0.05), and less out-of-pocket costs (β= -0.156, p < 0.001).

## Discussion

Diabetes is a complex and multi-causal disorder that is associated with considerable morbidity and mortality among patients and results in a heavy burden on healthcare resources. The global costs of diabetes are huge and will substantially increase, which calls for policymakers to take urgent actions to prepare health and social security systems to ensure affordable access to anti-diabetic drugs [60]. What’s more, thousands of new anti-diabetes drugs are synthesized each year. The reimbursement of new drugs that meet patient needs is of global interest. Meanwhile, the fulfillment of patients’ expectations of insurance benefits is the major predictor of satisfaction with health insurance [15]. Our study contributes to the understanding and incorporation of patient preferences into health insurance decision-making, thus informing policymakers to make coverage and reimbursement strategies more effective and resulting in higher patient satisfaction.

We found that patients’ preferred new anti-diabetic drugs for public health insurance coverage comprising the following attributes: improving patient-reported health status as reflected by HRQoL, bringing long-term health benefits, producing the expected treatment effects, causing few serious side effects, and having convenient dosing frequency. Patients’ preferences for multiple attribute values of new anti-diabetic drugs highlight the importance of systematic assessment and deliberate trade-offs of drugs during the decision-making process.

Among the attributes, health benefits defined as optimal HRQoL, extended life expectancy, and satisfied HbA_1c_ control were the most influential drivers of insurance coverage preferences. A systematic review showed that HRQoL was an essential attribute in the value-assessment framework for new medicines [61]. Length of extended life years represented the long-term benefits of the new drugs, while HbA_1c_ control was the primary outcome measure in evidence-based clinical practice guidelines. Improvements in health outcomes were also identified as the main factors that determined patients’ choices for anti-diabetic medicines in clinical practice [34, 62]. Accordingly, the effective interventions that can improve health outcomes can be included in the drug formulary and prioritized for reimbursement.

Our estimates of the main model also indicated that two attribute levels, the lowest incidence of serious side effects and the most convenient dosing, were statistically significant. However, dosing frequency was the least important attribute. Patients on average might be unwilling to trade quality of life and clinical benefits for convenience and safety. But our results were different from several previous studies. For example, a study found that diabetes patients in Germany and Spain were willing to trade efficacy for improvements in side effects [32]. Another study found that key determinants of treatment preferences among diabetes patients in Germany and the United Kingdom were side effects, efficacy, and dosing frequency [63]. It should be noted that only the HbA_1c_ level was used as an attribute of health benefits in those studies. In our study, not only the clinical outcomes like HbA_1c_ control but also attributes to reflect patient-reported outcomes (i.e., improvement in HRQoL) and long-term endpoints (i.e., extended life years) were involved. Meanwhile, we did not aim to assess patients’ preferences for therapeutic treatment; instead, we focused on the health insurance decisions.

To help policymakers better design health insurance schemes that satisfy individual patients’ needs, we explored preference heterogeneity. We had several new findings on the determinants of patients’ choices according to their demographic features. First, low-income patients were more concerned with the HbA_1c_ control, as well as less out-of-pocket costs. This is consistent with prior study findings that a disproportionately low rate of receiving anti-diabetic medication and having blood glucose monitoring was observed among the low-income patients in China [64, 65]. China’s universal health insurance coverage has been based on a strategy of wide coverage with a relatively low level of benefit, causing concerns about the limited financial protection offered by insurance programs, especially for the low-income populations [7]. Effective new drugs covered by public health insurance should be accessible to low-income patients at the most affordable costs.

Second, older patients particularly favored new drugs with a low incidence of serious adverse events and less out-of-pocket costs. Age has been identified as a risk factor for serious adverse events like severe hypoglycemia [66, 67]. We also found that younger adults cared more about the never or rarely incidence of serious adverse events, probably due to the relatively lower rate of adverse events among them. Our results inform insurance decision-makers to establish monitoring mechanisms for drug-related serious adverse events among older adults. The likelihood of morbidity and mortality increases with the aging process, which leads to a higher probability of out-of-pocket cost burden among older adults with diabetes [68]. The higher out-of-pocket costs, coupled with lower average incomes for older adults, might account for a higher economic burden than the younger adults [69]. Therefore, compared to the general population, it is necessary to further expand the reimbursement ratio of public health insurance for older adults, especially those with low incomes.

Finally, patients with diabetes complications expressed stronger preferences for the new drugs that contributed to the improvement of HRQoL, extended life years, and had less out-of-pocket costs. It has been proved that patients with complications have considerable impairment in HRQoL and suffered from life-year loss [70, 71]. Despite China’s efforts in improving its healthcare system, the financial burden for diabetes patients suffering from complications was still substantial, and some families became impoverished due to medical expenditures associated with the complications [72]. Likewise, the growing pressures on cost containment of rising healthcare expenditure require scrutiny and assessment of drugs for better value for patients with complications.

The major contributions of our DCE are as follows. First, we used a DCE which followed good research practices, offering the advantage to measure trade-offs in patient choices, quantify the strength of preferences and identify preference heterogeneity. Second, we involved attributes from clinical benefits, long-term endpoints, patient-reported outcomes, safety, convenience, and out-of-pocket costs. Our findings would be helpful for policymakers’ better understanding of the multi-attribute value of new anti-diabetic drugs. Third, we captured preference heterogeneity evidence to help policymakers make health insurance decisions more patient-centered.

Despite the strengths, several limitations of our study should be acknowledged. First, our samples were from Jiangsu province, which represents one of the most economically developed regions in China. Future studies should draw a nationally representative sample by including the economically underdeveloped regions. Second, we only selected a subset of prominent attributes that were identified from the literature review and focus group discussion. Our analysis did not address other attributes that might be meaningful to patients. Third, the role of private health insurance in extending universal health insurance coverage in China is limited at present. We are not sure whether the results are applicable to private health insurance schemes. Fourth, our study provides evidence of patients’ preferences for the multi-attribute value of new anti-diabetic drugs. Future studies are suggested to enroll patients with other types of diseases. Meanwhile, given the complexity of the health insurance system, we also recommend researchers to conduct studies to assess patients’ preferences for insurance plans, copayments, etc. Finally, DCEs pose hypothetical choices, which may not fully represent the choices respondents have or would make in real-world decision scenarios.

## Conclusion

In summary, our study showed that diabetes patients, in general, valued several attributes of new drugs, including effectiveness, patient-reported outcomes, economic consequences, safety, and convenience. The most influential drivers of patient preferences were health benefits like satisfied HRQoL, extended life years, and well-controlled HbA_1c_. Our findings also underline the value of accounting for preference heterogeneity in policy-making. Patient-centered public health insurance decision-making should be promoted, so as to enable the improved health outcomes and satisfaction of patients.

## Electronic supplementary material

Below is the link to the electronic supplementary material.

Supplementary Material 1

## Data Availability

Original datasets will be available upon reasonable request to the corresponding author.

## Abbreviations

HTA: Health technology assessment.
DCE: Discrete choice experiment.
HbA_1c_: Glycated haemoglobin A_1c_.
HRQoL: Health-related quality of life.
RI: Relative importance.
