# Supplementary material for: Investigating patients’ preferences for new anti-diabetic drugs to inform public health insurance coverage decisions: a discrete choice experiment in China
Source: BMC Public Health. 2022 Oct 5;22:1860. doi: 10.1186/s12889-022-14244-z (PMC9533494; doi:10.1186/s12889-022-14244-z)

**Appendix 1 Flow diagram for identification of the included DCEs**

Records identified through database searching (n=5326)

Records after duplicates removed (n=2590)

Full-text studies assessed for eligibility (n=66)

Full-text DCEs included

(n=30)

Records excluded after reading titles and abstracts (n=2524)

- Not relevant to diabetes (n=1491)
- Non-DCEs (n=879)
- Not relevant to topic (n=57)
- Non-original study (n=97)

Full-text studies excluded (n=36)

- Duplicate data (n=4)
- Not relevant to diabetes (n=3)
- Not relevant to topic (n=2)
- Non-DCEs (n=26)
- Unavailable data (n=1)

Duplicate records excluded (n=2736)

**Supplemental Figure 1** Flow diagram for identification of the included studies

*Notes:* Studies were identified by searching the following databases: PubMed, Embase, Web of Science, psycINFO, Cumulative Index to Nursing and Allied Health (CINAHL), Wanfang Data, VIP Chinese Science and technique Journals Database, and China Nation Knowledge (CNKI). Each database was searched from its inception to July 5, 2020. Reference lists from the retrieved documents were also searched.

Our search strategy mainly involved the following search terms: diabetes mellitus, blood glucose, blood glucose, HbA_1c_, type 2 diabetes, hyperglycemia, glycosylated hemoglobin; choice behavior, choice model, choice analysis, patient preference, preference-based method, preference-based approach, stated choice, stated preference, conjoint studies, conjoint analysis, conjoint measurement, conjoint choice experiment, discrete choice experiment, stated choice, pairwise comparison, multi-criteria decision analysis, multi-attribute decision analysis, multiple criteria decision making, multiple criteria decision analysis, MCDA, maximum difference scaling, best-worst scaling, analytic hierarchy process.

**Appendix 2 Characteristics of the included DCEs**

**Supplemental Table 1** Characteristics and attributes of the included DCEs

| **ID** | **Setting** | **Type of diabetes** | **Perspective** | **Attributes** | | | | | |
| --- | --- | --- | --- | --- | --- | --- | --- | --- | --- |
|  |  |  |  | **Effectiveness** | **Safety/ tolerability** | **Economic consequence** | **Patient-reported outcomes** | **Convenience** | **Others** |
| Aristides M 2004[1] | France/Germany/  Italy/Spain/UK | 2^$^ | Patient | √ | √ | √ | √ | √ | × |
| Guimarães C 2009[2] | Canada | 1^£^, 2 | Patient | √ | √ | √ | × | √ | × |
| Hauber AB 2009[3] | USA/UK | 2 | Patient | √ | √ | × | × | × | × |
| Jendle J 2010[4] | Sweden | 2 | Patient | √ | √ | √ | × | √ | √ |
| Bøgelund M 2011[5] | Denmark | 2 | Patient | √ | √ | √ | × | √ | √ |
| Casciano R 2011[6] | Africa/Middle East/Asia/  Europe/Latin America^#^ | 1, 2 | Patient | √ | × | × | × | √ | √ |
| Llyod A 2011[7] | UK | 1, 2 | Patient | √ | √ | √ | × | √ | × |
| Gelhorn HL 2013[8] | UK | 2 | Patient | √ | × | × | × | × | √ |
| Hauber AB 2013[9] | USA/UA | 2 | Patient | √ | √ | √ | × | √ | × |
| Mohamed AF 2013[10] | Sweden/Germany | 2 | Patient | √ | √ | √ | × | √ | × |
| Poulos C 2013[11] | USA/UK | 2 | Patient | √ | √ | × | √ | √ | √ |
| Veldwijk J 2014[12] | Dutch | 2 | Patient | × | √ | √ | √ | √ | × |
| Gelhorn HL2015[13] | Japan | 2 | Patient | √ | √ | √ | × | √ | × |
| Hauber AB 2015[14] | USA | 2 | Patient | √ | √ | √ | × | √ | × |
| Mol PG 2015 [15] | Dutch | 2 | Patient,  physician,  decision-maker | √ | √ | × | × | × | × |
| Morillas C 2015[16] | Spain/Portugal | 2 | Patient,  physician | √ | √ | √ | √ | √ | √ |
| Feher MD 2016[17] | North America/South America/Europe* | 2 | Patient | √ | √ | √ | × | √ | × |
| Hauber AB 2016[18] | USA | 2 | Patient | × | × | × | √ | √ | × |
| Mühlbacher A 2016[19] | Germany | 2 | Patient | √ | √ | √ | × | × | × |
| Ma L2016[20] | China | 1, 2 | Physician | × | × | √ | × | √ | × |
| Janssen EM 2017[21] | Spain | 2 | Patient | √ | √ | √ | × | √ | × |
| Mansfield C 2017[22] | Germany/Spain | 2 | Patient | √ | √ | × | × | √ | × |
| Qin L 2017[23] | Germany/UK | 2 | Patient | √ | √ | × | √ | √ | × |
| Fifer S 2018[24] | Australia | 2 | Patient | × | √ | √ | √ | √ | × |
| Forsander G 2018[25] | Sweden | 1 | Patient | √ | √ | × | × | √ | √ |
| Janssen EM 2018[26] | USA | 2 | Patient | √ | √ | √ | × | √ | × |
| Brooks A 2019[27] | Japan | 2 | Patient | √ | √ | × | × | √ | × |
| Marchesini G 2019[28] | Italian | 2 | Patient | × | √ | × | × | √ | × |
| Zhou M 2019[29] | USA | 2 | Patient | √ | √ | √ | × | √ | × |
| Donnan JR 2019[30] | Canada | 2 | Patient | √ | √ | √ | × | × | × |

*Notes:* ^£^Type 1 diabetes mellitus; ^$^Type 2 diabetes mellitus.

“√” meant that attributes were identified in DCEs, and in contrast, “×” meant that attributes were not identified in DCEs.

^#^Africa/Middle East (Algeria, Egypt, Iran, Lebanon, Morocco, Tunisia, Saudi Arabia, United Arab Emirates), Asia (China, Malaysia, Thailand), Europe (Turkey), Latin America (Argentina, Chile, Colombia, Guatemala, Mexico, Venezuela).

^*^ North America (USA, Canada), South America (Brazil, Chile, Colombia, Argentina, Mexico), Europe (UK, Germany, Netherlands, Italy, France).

**References**

**1.** Aristides M, Weston AR, FitzGerald P, Le Reun C, Maniadakis N. Patient preference and willingness-to-pay for humalog mix25 relative to humulin 30/70: a multicountry application of a discrete choice experiment. *Value Health.* 2004;7:442-454. <https://doi.org/10.1111/j.1524-4733.2004.74007.x>

**2.** Guimarães C, Marra CA, Colley L, et al. Socioeconomic differences in preferences and willingness-to-pay for insulin delivery systems in type 1 and type 2 diabetes. *Diabetes Technol The.* 2009;11:567-573. <https://doi.org/10.1089/dia.2009.0034>

**3.** Hauber AB, Mohamed AF, Johnson FR, Falvey H. Treatment preferences and medication adherence of people with type 2 diabetes using oral glucose-lowering agents. *Diabetic Med.* 2009;26:416-424. <https://doi.org/10.1111/j.1464-5491.2009.02696.x>

**4.** Jendle J, Torffvit O, Ridderstrale M, Lammert M, Ericsson A, Bogelund M. Willingness to pay for health improvements associated with anti-diabetes treatments for people with type 2 diabetes. *Curr Med Res Opin.* 2010;26:917-923. <https://doi.org/10.1185/03007991003657867>

**5.** Bøgelund M, Vilsbøll T, Faber J, Henriksen JE, Gjesing RP, Lammert M. Patient preferences for diabetes management among people with type 2 diabetes in Denmark a discrete choice experiment. *Curr Med Res Opin.* 2011;27:2175-2183. <https://doi.org/10.1185/03007995.2011.625404>

**6.** Casciano R, Malangone E, Ramachandran A, Gagliardino JJ. A quantitative assessment of patient barriers to insulin. *Int J Clin Pract.* 2011;65:408-414. <https://doi.org/10.1111/j.1742-1241.2010.02590.x>

**7.** Lloyd A, Nafees B, Barnett AH, et al. Willingness to pay for improvements in chronic long-acting insulin therapy in individuals with type 1 or type 2 diabetes mellitus. *Clin Ther.* 2011;33:1258-1267. <https://doi.org/10.1016/j.clinthera.2011.07.017>

**8.** Gelhorn HL, Stringer SM, Brooks A, et al. Preferences for medication attributes among patients with type 2 diabetes mellitus in the UK. *Diabetes Obes Metab.* 2013;15:802-809. <https://doi.org/10.1111/dom.12091>

**9.** Hauber AB, Han S, Yang JC, et al. Effect of pill burden on dosing preferences, willingness to pay, and likely adherence among patients with type 2 diabetes. *Patient Prefer Adher.* 2013;7:937-949. <https://doi.org/10.2147/PPA.S43465>

**10.** Mohamed AF, Zhang J, Johnson FR, et al. Avoidance of weight gain is important for oral type 2 diabetes treatments in Sweden and Germany: patient preferences. *Diabetes Metab.* 2013;39:397-403. <https://doi.org/10.1016/j.diabet.2013.06.001>

**11.** Poulos C, González JM, Lee LJ, et al. Physician preferences for extra-glycemic effects of type 2 diabetes treatments. *Diabetes Ther.* 2013;4:443-459. <https://doi.org/10.1007/s13300-013-0046-7>

**12.** Veldwijk J, Lambooij MS, De Bekker-Grob EW, Smit HA, De Wit GA. The effect of including an opt-out option in discrete choice experiments. *PLoS One.* 2014;9:e111805. <https://doi.org/10.1371/journal.pone.0111805>

**13.** Gelhorn HL, Poon JL, Davies EW, Paczkowski R, Curtis SE, Boye KS. Evaluating preferences for profiles of GLP-1 receptor agonists among injection-naïve type 2 diabetes patients in the UK. *Patient Prefer Adher.* 2015;9:1611-1622. <http://doi.org/10.2147/ppa.S90842>

**14.** Hauber AB, Tunceli K, Yang JC, et al. A survey of patient preferences for oral antihyperglycemic therapy in patients with type 2 diabetes mellitus. *Diabetes Ther.* 2015;6:75-84. <https://doi.org/10.1007/s13300-015-0094-2>

**15.** Mol PG, Arnardottir AH, Straus SM, et al. Understanding drug preferences, different perspectives. *Br J Clin Pharmacol.* 2015;79:978-987. <https://doi.org/10.1111/bcp.12566>

**16.** Morillas C, Feliciano R, Catalina PF, et al. Patients’ and physicians’ preferences for type 2 diabetes mellitus treatments in Spain and Portugal: a discrete choice experiment. *Patient Prefer Adher.* 2015;9:1443-1458. <https://doi.org/10.2147/PPA.S88022>

**17.** Feher MD, Brazier J, Schaper N, Vega-Hernandez G, Nikolajsen A, Bøgelund M. Patients’ with type 2 diabetes willingness to pay for insulin therapy and clinical outcomes. *BMJ Open Diab Res Ca.* 2016;4:e000192. <https://doi.org/10.1136/bmjdrc-2016-000192>

**18.** Hauber AB, Nguyen H, Posner J, Kalsekar I, Ruggles J. A discrete-choice experiment to quantify patient preferences for frequency of glucagon-like peptide-1 receptor agonist injections in the treatment of type 2 diabetes. *Curr Med Res Opin.* 2016;32:251-262. <https://doi.org/10.1185/03007995.2015.1117433>

**19.** Mühlbacher A, Bethge S. What matters in type 2 diabetes mellitus oral treatment? A discrete choice experiment to evaluate patient preferences. *Eur J Health Econ.* 2016;17:1125-1140. <https://doi.org/10.1007/s10198-015-0750-5>

**20.** Ma L, Han S, Guan XD, Shi LW, Li W. Investigation and research of endocrinologists’ prescription preferences of insulin injection pen in China: based on discrete choice experiment. *China Academic Electronic Publishing House* 2016;27:5055-5058.

**21.** Janssen EM, Longo DR, Bardsley JK, Bridges JFP. Education and patient preferences for treating type 2 diabetes: a stratified discrete-choice experiment. *Patient Prefer Adher.* 2017;11:1729-1736. <https://doi.org/10.2147/PPA.S139471>

**22.** Mansfield C, Sikirica MV, Pugh A, et al. Patient preferences for attributes of type 2 diabetes mellitus medications in Germany and Spain: an online discrete-choice experiment survey. *Diabetes Ther.* 2017;8:1365-1378. <https://doi.org/10.1007/s13300-017-0326-8>

**23.** Qin L, Chen S, Flood E, et al. Glucagon-like peptide-1 receptor agonist treatment attributes important to injection-experienced patients with type 2 diabetes mellitus: a preference study in Germany and the United Kingdom. *Diabetes Ther.* 2017;8:335-353. <https://doi.org/10.1007/s13300-017-0237-8>

**24.** Fifer S, Rose J, Hamrosi KK, Swain D. Valuing injection frequency and other attributes of type 2 diabetes treatments in Australia: a discrete choice experiment. *BMC Health Serv Res.* 2018;18:675. <https://doi.org/10.1186/s12913-018-3484-0>

**25.** Forsander G, Stallknecht S, Samuelsson U, Marcus C, Bøgelund M. Preferences for treatment among adolescents with Type 1 diabetes: a national study using a discrete choice experiment model. *Diabetic Med.* 2018;35:621-629. <https://doi.org/10.1111/dme.13592>

**26.** Janssen EM, Hauber AB, Bridges JFP. Conducting a discrete-choice experiment study following recommendations for good research practices: an application for eliciting patient preferences for diabetes treatments. *Value Health.* 2018;21:59-68. <https://doi.org/10.1016/j.jval.2017.07.001>

**27.** Brooks A, Langer J, Tervonen T, Hemmingsen MP, Eguchi K, Bacci E. Patient preferences for glp-1 receptor agonist treatment of type 2 diabetes mellitus in Japan: a discrete choice experiment. *Diabetes Ther.* 2019;10:735-749. <https://doi.org/10.1007/s13300-020-00766-5>

**28.** Marchesini G, Pasqualetti P, Anichini R, et al. Patient preferences for treatment in type 2 diabetes: the Italian discrete-choice experiment analysis. *Acta Diabetol.* 2019;56:289-299. <https://doi.org/10.1007/s00592-018-1236-6>

**29.** Zhou M, Bridges JFP. Explore preference heterogeneity for treatment among people with type 2 diabetes: a comparison of random-parameters and latent-class estimation techniques. *J Choice Model.* 2019;30:38-49. <https://doi.org/10.1016/j.jocm.2018.11.002>

**30.** Donnan JR, Johnston K, Chibrikov E, et al. Capturing adult patient preferences toward benefits and risks of second-line antihyperglycemic medications used in type 2 diabetes: a discrete choice experiment. *Can J Diabetes.* 2020;44:6-13. <https://doi.org/10.1016/j.jcjd.2019.04.014>

**Appendix 3 Explanations of the study attributes and levels**

Investigators were required to convey the following definitions to patients:

- ‘HbA_1c_ control’ means that the ideal treatment goals of HbA_1c_ set out in the evidence-based guidelines for individual patients can be achieved. *‘Not as expected’* implies that there can be certain treatment effects while the HbA_1c_ targets cannot be fully achieved; *‘As expected’* implies that the ideal treatment effects can be fully achieved. This attribute represents the short-term health benefits.
- ‘Serious side effects’ refers to drug-related serious adverse events. The events are life-threatening, and patients need to receive medical care immediately. Serious adverse events such as coma caused by severe hypoglycemia, serious damage to the liver, kidney, and other organs. *‘Sometimes’* is equivalent to the incidence of serious side effects approximately 5%; *‘Occasionally’* equals 2%; ‘Never or rarely’ means 1‰ or less.
- ‘Length of extended life’ means extension of life years after taking drugs, theoretically. This attribute indicates the long-term health benefits.
- ‘Change in HRQoL’ represents long-term benefit of the comparative patient-reported outcomes. This attribute could capture the patients’ perspective regarding the impact of new drugs on their lives. *‘Worse’* means that patients’ HRQoL become worse after treatment. *‘No improvement’* means that patients’ HRQoL remain the status of quo compared with pre-treatment. *‘Improvement’* shows that patients’ HRQoL have improvement significantly.
- ‘Dosing frequency’ refers to the frequency of taking drugs. This attribute represents the convenience of new drugs.
- ‘Out-of-pocket costs’ per month refers to the monthly amount a patient has to pay after getting reimbursed from the public health insurance programs. This attribute reflects the economic consequences of new drugs.

**Appendix 4 Examples of DCE choice sets**

Suppose your blood glucose is out of control, and you need a new drug to treat diabetes. There are two types of new drugs, drugs A and B. They have not been reimbursed by public health insurance programs. If only one drug could be covered due to the constrained budget of health insurance, which one would you prefer? Please think carefully and make a trade-off between the two new drugs.

| **Attributes** | **New therapeutic drug A** | **New therapeutic drug B** |
| --- | --- | --- |
| Out-of-pocket costs per month (if reimbursed) | 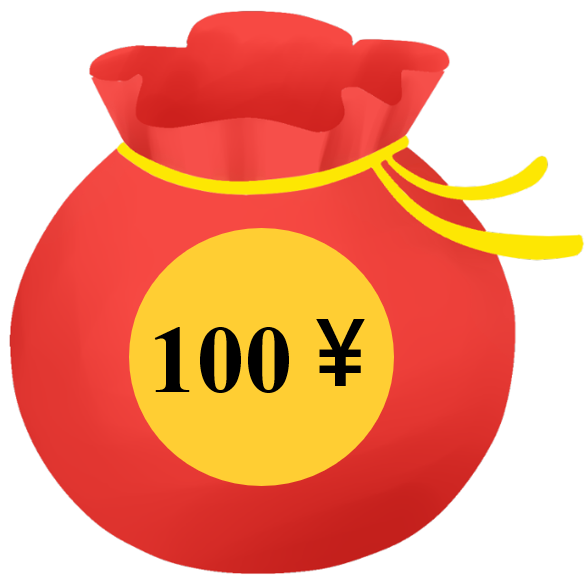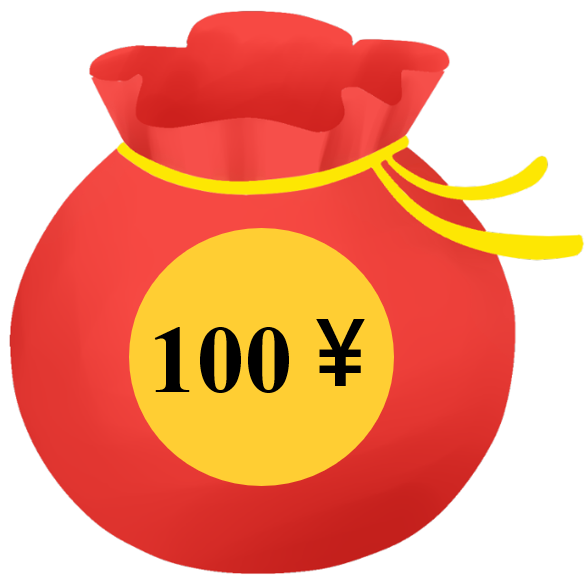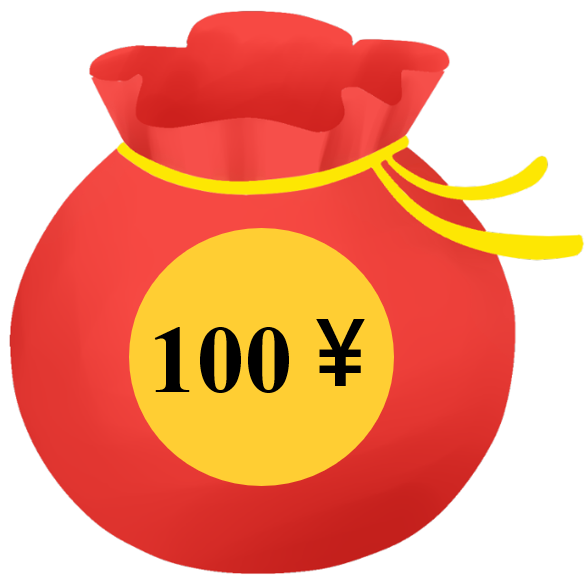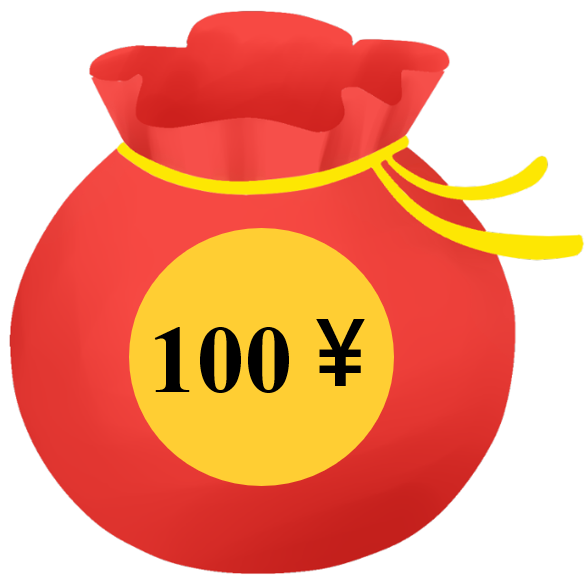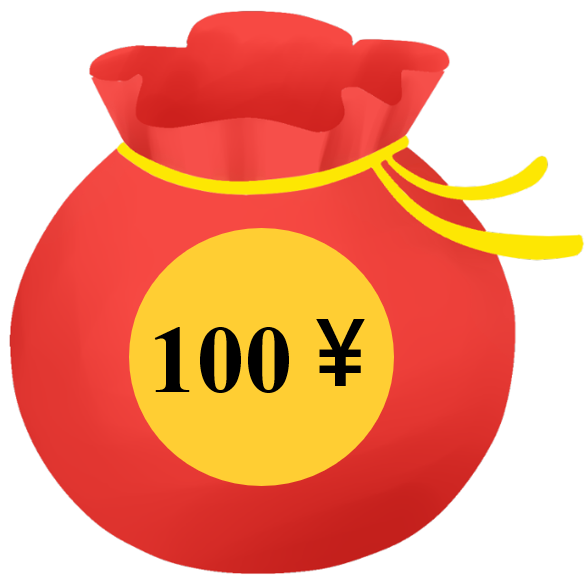  CNY 500 per month | 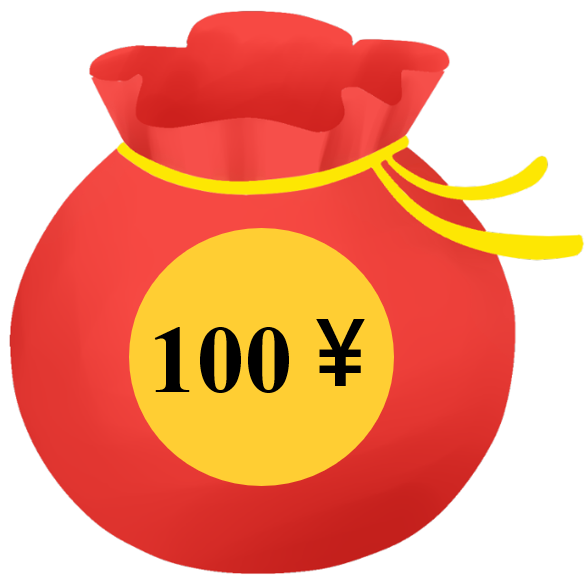  CNY 100 per month |
| HbA_1c_ control | 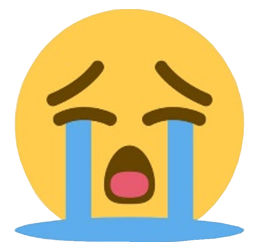  Not as excepted | 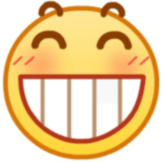  As excepted |
| Serious side effects  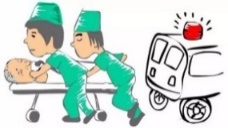  (Life-threatening, need to see a doctor) | 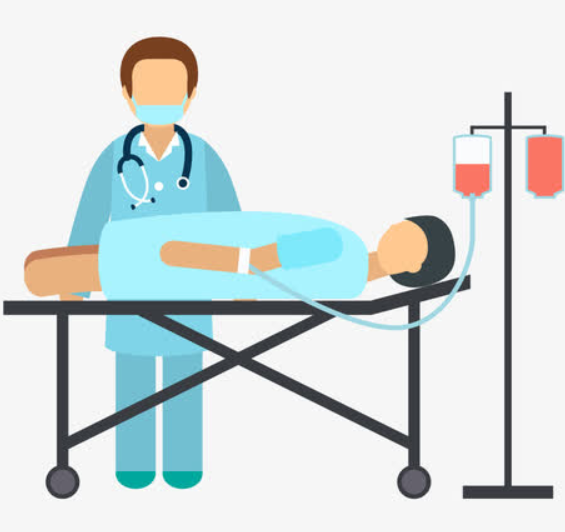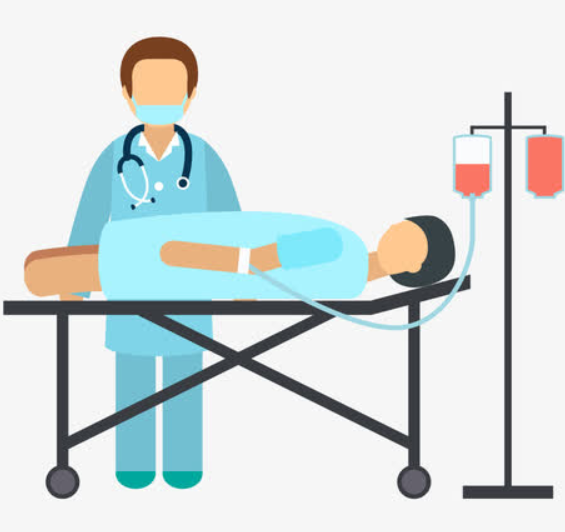  Occasionally  (2%) | 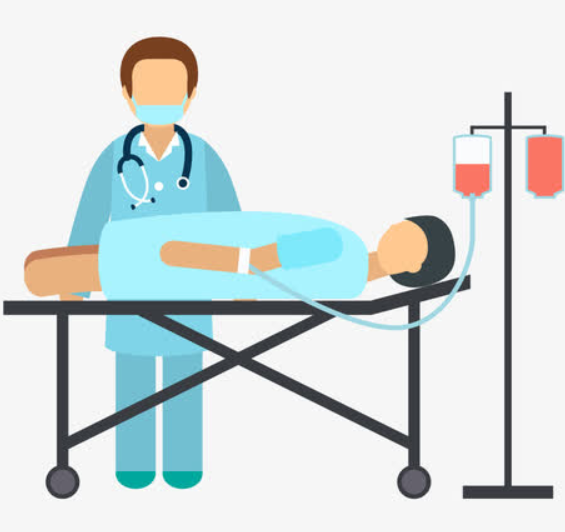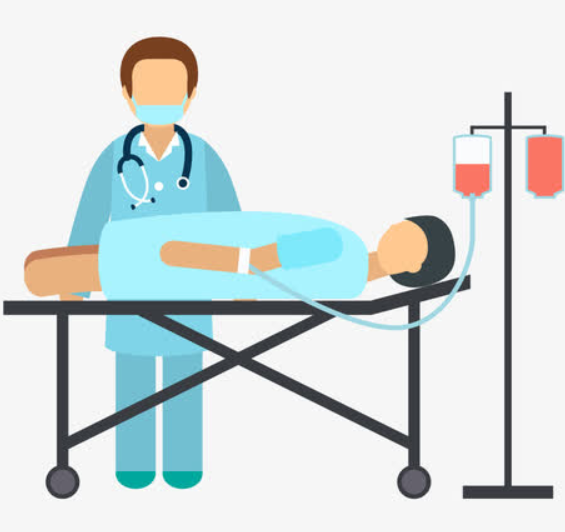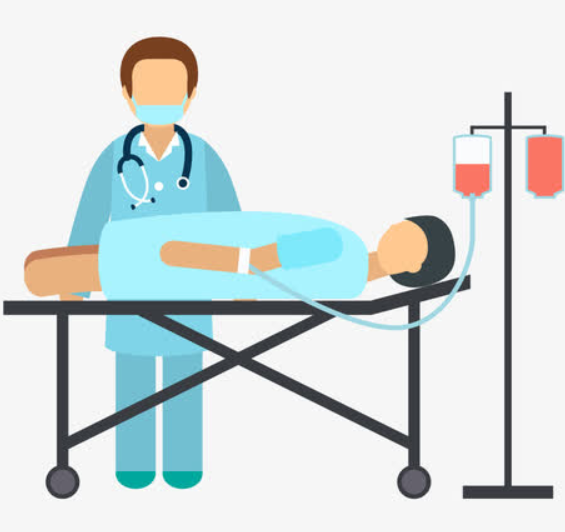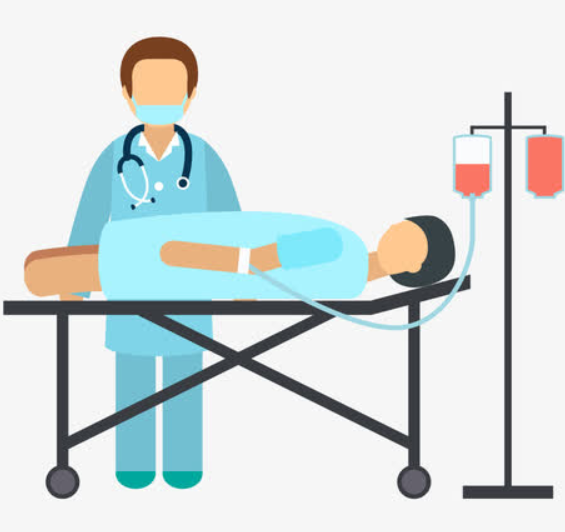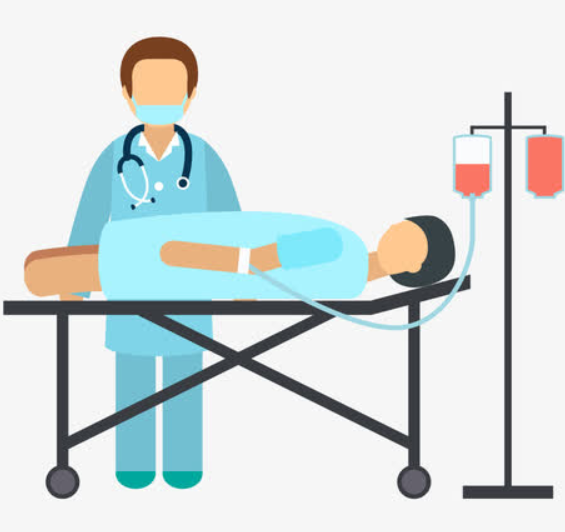  Sometimes  (5%) |
| Length of extended life  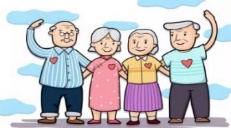 | 0.5 years | 3.5 year |
| Change in HRQoL | 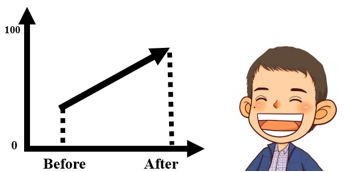  Improvement | 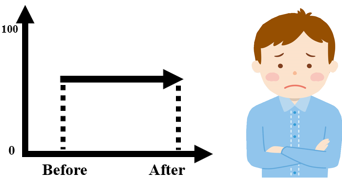  No improvement |
| Dosing frequency | 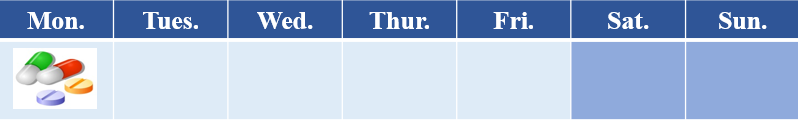  Once a week | 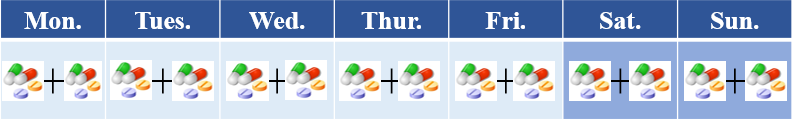  Twice a day |
| Which one should be covered by health insurance? Your choice | 🞏 | 🞏 |

**Appendix 5 Evaluation of patients’ understanding and confidence in DCE choices**

1. Do you feel difficult or easy to understand the DCE scenarios and choice sets? Please select a level from zero to 10 and give a tick ‘√’ in the score to reflect your understanding:

0: extremely

difficult

10: extremely

easy


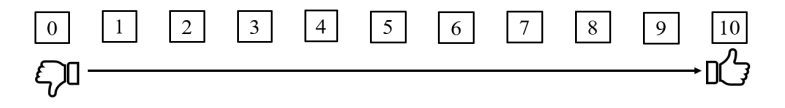


2. Are you confident in your choice of the new drug? Please select a level from zero to 10 and give a tick ‘√’ in the score to represent your confidence:

10: extremely confident

0: not confident

at all


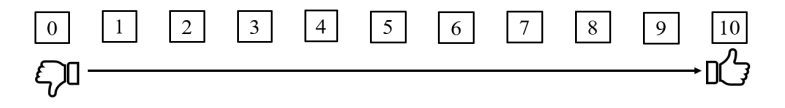

Supplement: Supplementary file 1 — Supplementary Material 1 [file 12889_2022_14244_MOESM1_ESM.docx]
